# Development of a novel program for conversion from tetrahedral‐mesh‐based phantoms to DICOM dataset for radiation treatment planning: TET2DICOM

**DOI:** 10.1002/acm2.13448

**Published:** 2021-10-11

**Authors:** Bo‐Wi Cheon, Se Hyung Lee, Min Cheol Han, Chul Hee Min, Haegin Han, Chan Hyeong Kim, Jin Sung Kim

**Affiliations:** ^1^ Department of Radiation Convergence Engineering Yonsei University Wonju Korea; ^2^ Department of Nuclear Engineering Hanyang University Seoul Korea; ^3^ Department of Radiation Oncology Bundang Jesaeng General Hospital Seongnam Korea; ^4^ Department of Radiation Oncology Yonsei University College of Medicine Seoul Korea

**Keywords:** clinical software, CT, DICOM, tetrahedral‐mesh phantom, voxelization

## Abstract

**Purpose:**

Tetrahedral mesh (TM)–based computational human phantoms have recently been developed for evaluation of exposure dose with the merit of precisely representing human anatomy and the changing posture freely. However, conversion of recently developed TM phantoms to the Digital Imaging and Communications in Medicine (DICOM) file format, which can be utilized in the clinic, has not been attempted. The aim of this study was to develop a technique, called TET2DICOM, to convert the TM phantoms to DICOM datasets for accurate treatment planning.

**Materials and methods:**

The TM phantoms were sampled in voxel form to generate the DICOM computed tomography images. The DICOM‐radiotherapy structure was defined based on the contour data. To evaluate TET2DICOM, the shape distortion of the TM phantoms during the conversion process was assessed, and the converted DICOM dataset was implemented in a commercial treatment planning system (TPS).

**Results:**

The volume difference between the TM phantoms and the converted DICOM dataset was evaluated as less than about 0.1% in each organ. Subsequently, the converted DICOM dataset was successfully implemented in MIM (MIM Software Inc., Cleveland, USA, version 6.5.6) and RayStation (RaySearch Laboratories, Stockholm, Sweden, version 5.0). Additionally, the various possibilities of clinical application of the program were confirmed using a deformed TM phantom in various postures.

**Conclusion:**

In conclusion, the TM phantom, currently the most advanced computational phantom, can be implemented in a commercial TPS and this technique can enable various TM‐based applications, such as evaluation of secondary cancer risk in radiotherapy.

## 1 INTRODUCTION

Modern radiotherapy (RT) techniques, such as intensity modulated radiation therapy and volumetric modulated arc therapy, have been the main means employed in external‐beam RT to direct a conformal dose distribution to tumor volumes while minimizing the dose to at‐risk organs.^1^ During treatment, these modalities deliver the dose to the target volume in a number of beam fractions. However, such hyper‐fractionation in beam delivery incurs 3–5 times more out‐of‐field dose due to dose leakage or scattered radiation than conventional techniques do.^2^, ^3^ It is important to consider the out‐of‐field dose in treatment planning, since it can cause side effects such as secondary cancer, cardiovascular disease, and hereditarily transmitted genetic defects.^4^, ^5^, ^6^

With the advancement of technology, the commercial treatment planning system (TPS) can accurately predict the dose of the organ at risk or normal tissue, but the accuracy of dose prediction may be lower as the distance from the in field is increased,^7^, ^8^ because the dose calculation engine of TPS is focused on the in‐field dose. Howell et al.^9^ and Huang et al.^10^ evaluated the calculation accuracy in case of out‐of‐field dose. The results show that additional studies are needed to accurately calculate the out‐of‐field dose, and that the out‐of‐field dose can be underestimated by up to 50%, respectively. In particular, in case of small organs, such as the prostate, a dose calculation error of up to about 40% may occur as the distance from the edge of the in field increases.^8^ Besides, the TPS is constructed with sufficient computed tomography (CT) images to be treated for an RT plan, but there may not be enough information on the patient's body to predict the low dose of out of field. To overcome this limitation, several studies have been carried out on the implementation, in the TPS, of a voxel‐based computational human phantom for the whole body by supplementing CT images’ insufficient information. Lee et al.^11^ first introduced a method to convert whole‐body human phantoms to Digital Imaging and Communications in Medicine (DICOM) CT images for use in the TPS. Lee et al.^12^ and Griffin et al.^13^ developed software for implanting voxel‐based computational human phantoms into the DICOM‐RT dataset (CT images and RT structure). They confirmed that the DICOM dataset thus converted could be clinically used in the TPS for evaluation of out‐of‐field dose.

However, voxel‐based computational human phantoms have had several limitations. As the voxel‐based computational phantom has a rigid structure, there is a problem in matching patient‐specific postures in clinics.^14^ Certainly, accurate evaluation of dose distributions, including the out‐of‐field dose, requires careful consideration of patient posture. Total body irradiation (TBI) and total skin electron beam therapy (TSEBT) results are highly influenced by patient posture.

Recently, several investigators have developed NURBS/polygonal mesh (PM)–based hybrid phantom that can address the limitations of the voxel phantom and be deformed in various postures.^12^, ^15^, ^16^, ^17^, ^18^ Based on this, the deformed hybrid phantom has been modeled with various ages and shapes. In addition, a program to convert the hybrid phantom to DICOM was developed for the purpose of clinical application.^11^ Although the hybrid phantoms, excluding NURBS phantom, can be implemented in the Monte Carlo (MC) simulation code, there are various limitations. It normally needs more computation speed than voxel phantom to implement in MC code^15^ and cannot represent inhomogeneous mass density distribution in deformed posture phantoms.^14^, ^19^

Helpfully though, tetrahedral mesh (TM)–based computational human phantoms were recently developed for the purposes of precise representation of human anatomy and subsequent evaluation of exposure dose in the radiation protection context.^14^, ^20^, ^21^ Indeed, the TM phantoms have been reported to be highly flexible for effective posture deformation,^22^ and micron‐scale radiosensitive tissues, such as the skin layer,^23^ eye lens,^24^ and Gastrointestinal (GI) tract,^25^ can be defined in TM phantom. In addition, the TM phantom has been selected as an international standard phantom and has been used in various studies.^21^ Therefore, the DICOM dataset converted from TM phantom makes it possible to calculate even in case of very thin organs, which was difficult in hybrid phantoms, and can be used in various studies through comparison with the MC simulation calculation results.

In this study, we developed a new C++ language‐based program, named TET2DICOM, for importation of the TM phantoms into the clinical DICOM dataset. To verify TET2DICOM, it was used to convert the reference TM phantoms to the DICOM dataset, and then the geometries of the converted DICOM CT images were compared with those of the original TM phantoms. Finally, to evaluate the clinical applicability of TET2DICOM, the converted DICOM dataset was implemented in the conventional TPS.

## 2 MATERIALS AND METHODS

### 2.1 Overview of TET2DICOM

**FIGURE 1:**
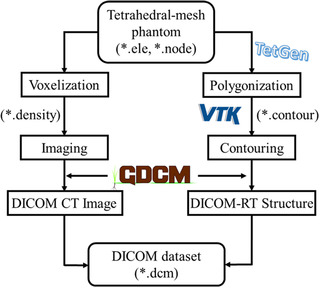
Workflow of TET2DICOM program to convert tetrahedral‐mesh phantom to Digital Imaging and Communications in Medicine dataset

The TM phantom is composed of a node file and an element file, which include the coordinates of the tetrahedral vertices and information on their connections, respectively. As illustrated in Figure 1, TET2DICOM operates in two independent processes to import the TM phantom to the DICOM dataset. Note that the developed program can comprehensively perform in a single run without the need for any additional user's manual, using the following processes: (1) CT image generation: the mass‐density of each tetrahedron for calculation of the Hounsfield units (HU) and (2) DICOM‐RT structure generation: user‐defined contours of the organs of interest.

### 2.2 Conversion of TM phantom to a DICOM CT image set

The DICOM CT set consists of patient‐specific metadata such as SOP Instance UID, Pixel Spacing, and Pixel Data. TET2DICOM employs the Grassroots DICOM library (GDCM, ver. 2.6)^26^ to generate DICOM metadata related to the CT image, such as the voxel data, the field of view (FOV), and the phantom's geometrical information. Notably, the voxel data are filled with HU values converted from predefined organ densities.

**FIGURE 2:**
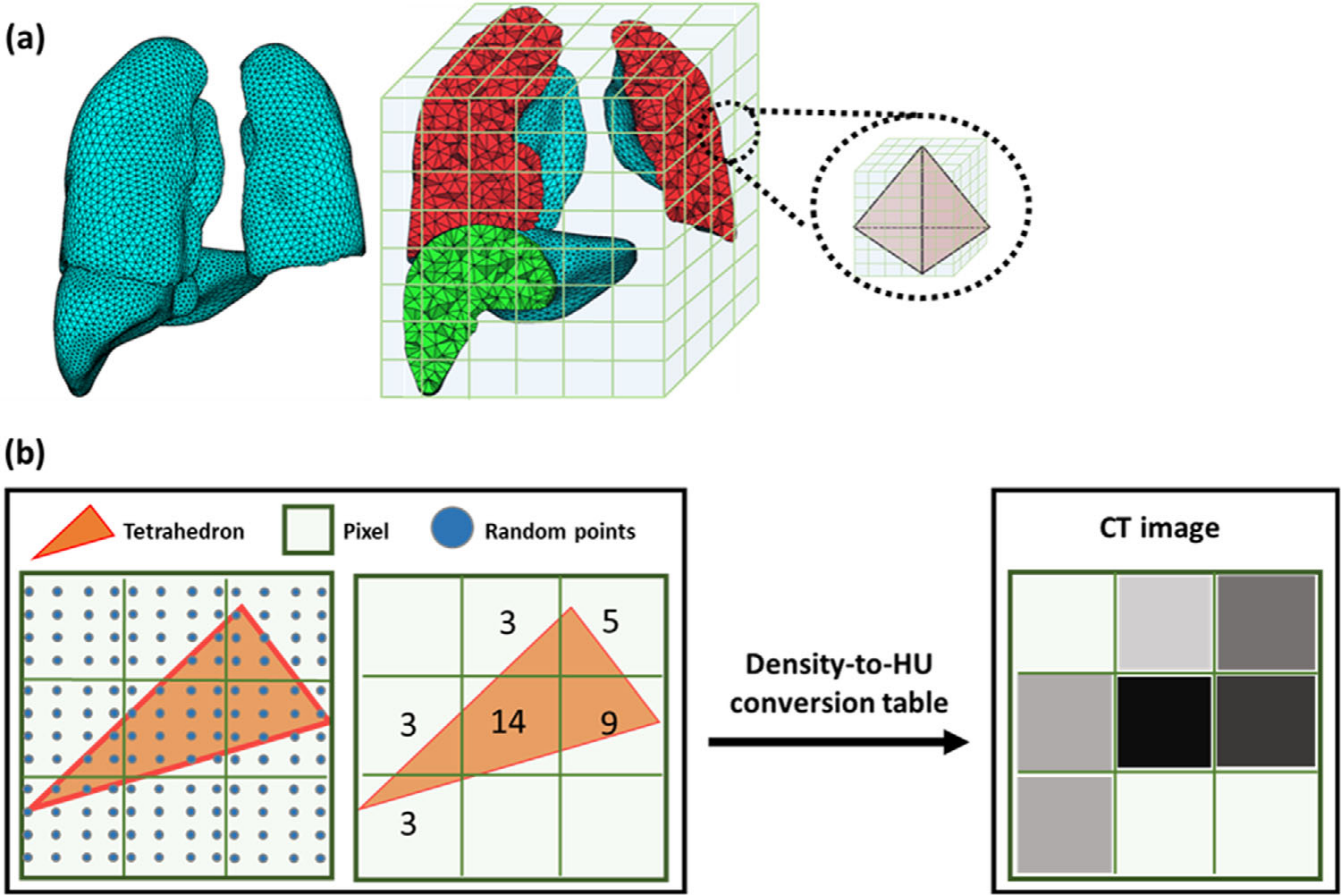
Example of computed tomography (CT) image generation: (a) tetrahedral‐mesh phantom for lungs and liver, and its virtual voxel and (b) generation of two‐dimensional CT image

The procedure of the conversion of the TM phantom into the DICOM CT image set is initiated from the voxelization of the TM phantom. As the first step of the voxelization process, the minimum and maximum values on coordinates are obtained by obtaining the coordinates of the tetrahedron constituting each organ. Afterward, based on the coordinate values, a cuboid‐shaped virtual voxel including the corresponding organ is generated. The created virtual voxel is divided into subvoxels according to the pixel size and pixel resolution defined by the user to create the virtual voxel grid (Figure 2a). The user can change the voxel size and pixel resolution, the default values of which are set as 0.976562 × 0.976562 × 2 mm^3^ and 512 × 512, respectively. The FOV can also be defined by the user; the default value is set as 500 × 500 mm^2^. The density in each virtual voxel ($D_{V}$) is calculated by considering the contribution to the voxel volume of each tetrahedron, using Equation (1):

(1) $$D_{V}=\sum_{i=1}^{n}w_{i}\times D_{tet,i}$$

In the equation, n is the number of tetrahedrons that contribute to the voxel volume. $W_{i}$ represents the weighting factor, defined as the ratio between the number of generated random points in tetrahedron i and the total number of random points in a voxel (Figure 2b). A thousand random points were produced in 1 mm^3^ volume to define the contribution of each tetrahedron to the voxel volume. $D_{tet,i}$ is the density of tetrahedron i. To determine whether the generated random points are positioned in or outside of a tetrahedron, the *Inside* function of G4Tet in Geant4 is employed. As shown in Figure 2b, the HU value for each voxel is defined by using the voxel density and the density‐to‐HU conversion table. Finally, the calculated HU values are inserted into the Pixel Data to generate the CT image set. The voxelization process was designed by using the OpenMP‐based multiprocess,^27^ because this process can effectively utilize huge computational resources. The voxel grid for the TM phantom is automatically saved when the voxelization is completed, so as to avoid repeating the voxelization for the same phantom.

### 2.3 Production of DICOM‐RT structure

**FIGURE 3:**
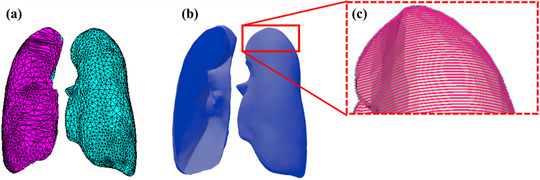
Example of contour points generation from tetrahedral‐mesh (TM) phantom: (a) TM phantom, (b) converted polygonal‐mesh (PM) phantom, and (c) generated contour points in PM phantom

A contour file includes a user‐defined list of the organs of interest generated with TET2DICOM. The contour file consists of three different data points, which are the number of organs, the organ ID, and the organ name. To extract the TM phantom's contour information, the original TM phantom (Figure 3a) is converted, using a software called TetGen,^28^ ^,^ ^29^ to a PM phantom that consists only of surfaces, as represented in Figure 3b. Then, the PM phantom is sliced into transverse planes, using the *vtkPlane* class in the Visualization Toolkit (VTK, ver. 8.1),^30^ in order to produce tomographic images. In Figure 3c, the red lines represent the connected contour points between the slice planes and PM phantoms. The contour data are converted to contour metadata using the GDCM in the same manner in which CT image files are created.

The DICOM‐RT structure file shares the common DICOM tags of the DICOM CT image, but there are essential tags that additionally need to be defined.^13^ In the TET2DICOM program, these essential tags are automatically generated through the GDCM. Additionally, the DICOM CT image set and the DICOM‐RT structure is merged into one DICOM set using the SOPInstanceUID tag.

### 2.4 Evaluation of the TM‐based DICOM dataset obtained by TET2DICOM

TET2DICOM was evaluated by producing the DICOM dataset from four different TM phantoms: the mesh‐type reference computational male and female phantoms (MRCP‐AM and MRCP‐AF, respectively)^15^ and the mesh‐type reference Korean male and female phantoms (MRKP‐AM and MRKP‐AF, respectively).^31^ All of the TM phantoms consisted of 48 organs. The number of tetrahedrons was approximately 8×10^6^ and 7 ×10^6^ for the MRCPs and MRKPs, respectively. Table 1 shows the parameters employed in the production of the DICOM CT images for each TM phantom. In this study, a workstation equipped with Intel(R) Xeon(R) Gold 5218R CPU (Twenty‐Core Processor 2.10 GHz and 192 GB RAM) was used in the production of the DICOM dataset.

**TABLE 1:** Parameters for production of computed tomography (CT) image with tetrahedral‐mesh (TM) phantoms

	CT image parameters				
Types of TM phantoms	Field of view (mm)	Pixel size (mm^2^)	Pixel resolution	Slice thickness (mm)	Number of slices
MRCP‐AM^1^	600	1.17× 1.17	512 ×512	2	882
MRCP‐AF^2^	600	1.17 ×1.17	512 ×512	2	834
MRKP‐AM^3^	600	1.17× 1.17	512×512	2	862
MRKP‐AF^4^	500	0.97×0.97	512 ×512	2	798

^1^ Mesh‐type reference computational male phantom

^2^ Mesh‐type reference computational female phantom

^3^ Mesh‐type reference Korean male phantom

^4^ Mesh‐type reference Korean female phantom

The DICOM datasets were evaluated by comparing the sectional images of the produced CT to the original TM phantoms. The shape of each organ was compared between the CT images and the TM phantoms in terms of the geometrical distortion that had occurred during the conversion. Subsequently, the DICOM datasets were imported to the TPS, and various postures were applied to the phantom for evaluation of TET2DICOM's clinical applicability.

## 3 RESULTS AND DISCUSSION

### 3.1 Conversion of TM phantoms

**FIGURE 4:**
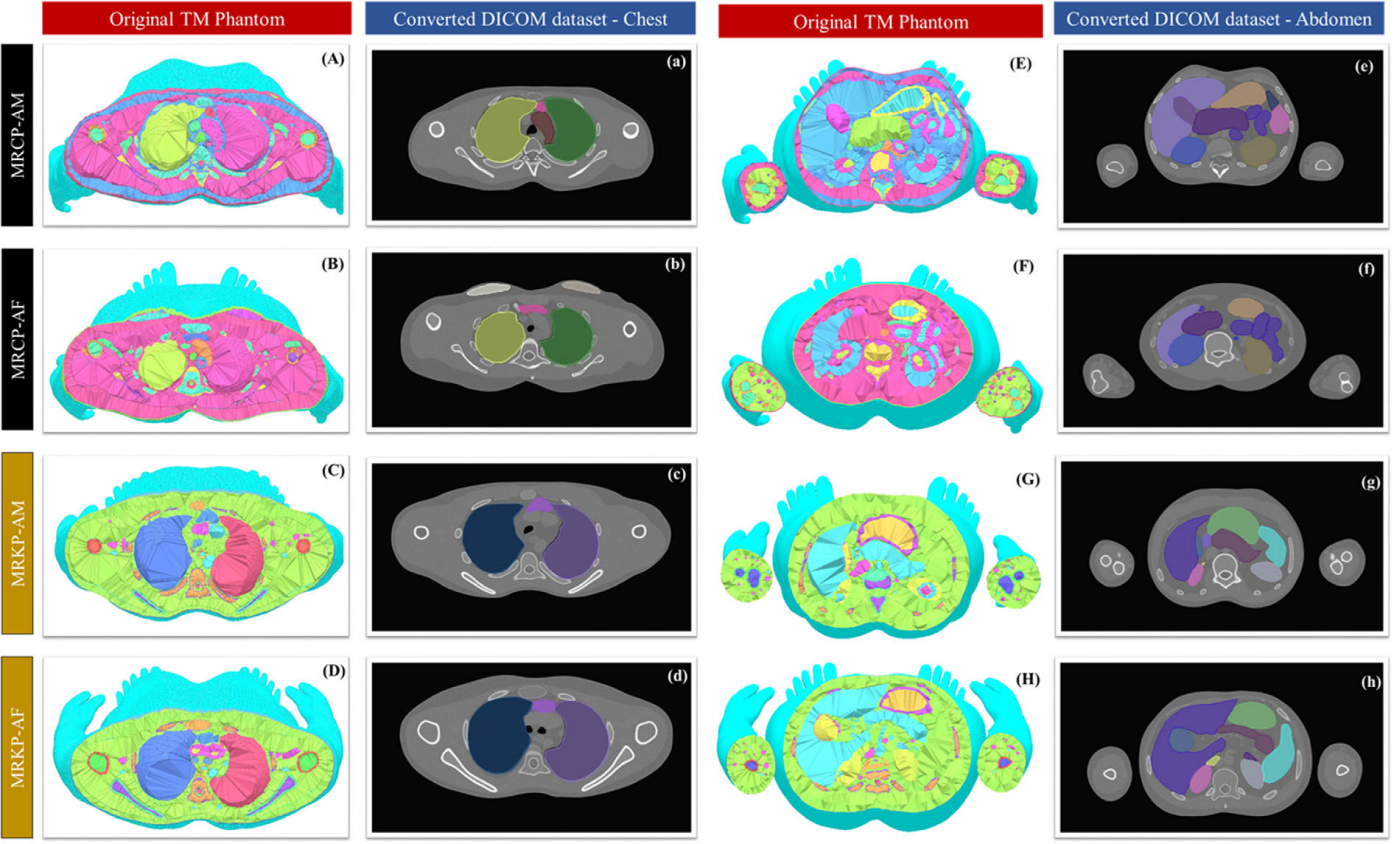
Transverse view of tetrahedral‐mesh phantoms and their Digital Imaging and Communications in Medicine datasets: (A–a, E–e) mesh‐type reference computational male, (B–b, F–f) mesh‐type reference computational female, (C–c, G–g) mesh‐type reference Korean male, and (D–d, H–h) mesh‐type reference Korean female

All of the pixel sizes were automatically determined by the FOV that the user could define. The number of slices generated for the phantoms ranged from 798 to 882, depending on the phantom's height. Figure 4 shows transverse views of the TM phantoms and DICOM CT images. As shown in Figure 4, it was confirmed that all of the TM phantoms were successfully imported to the DICOM dataset with no geometrical distortion, and qualitatively checked to make sure that the external body shape and internal organs of the TM phantoms were clearly defined in the CT image.

TET2DICOM took 100–121 min to produce the DICOM dataset in this study, and most of the runtime was occupied by the voxelization process. More computation time was required with a larger number of tetrahedrons in the TM phantom. For example, there was a need for about 460 mins to convert all organs except the skin in the MRCP‐AM phantom to the DICOM dataset. The voxelization log files were saved when the TM phantom was converted for the first time, reusing the log file for reconversion. With respect to the reconversion procedure, the conversion time ends within a few minutes.

### 3.2 Implementation of the converted DICOM dataset in clinical software

**FIGURE 5:**
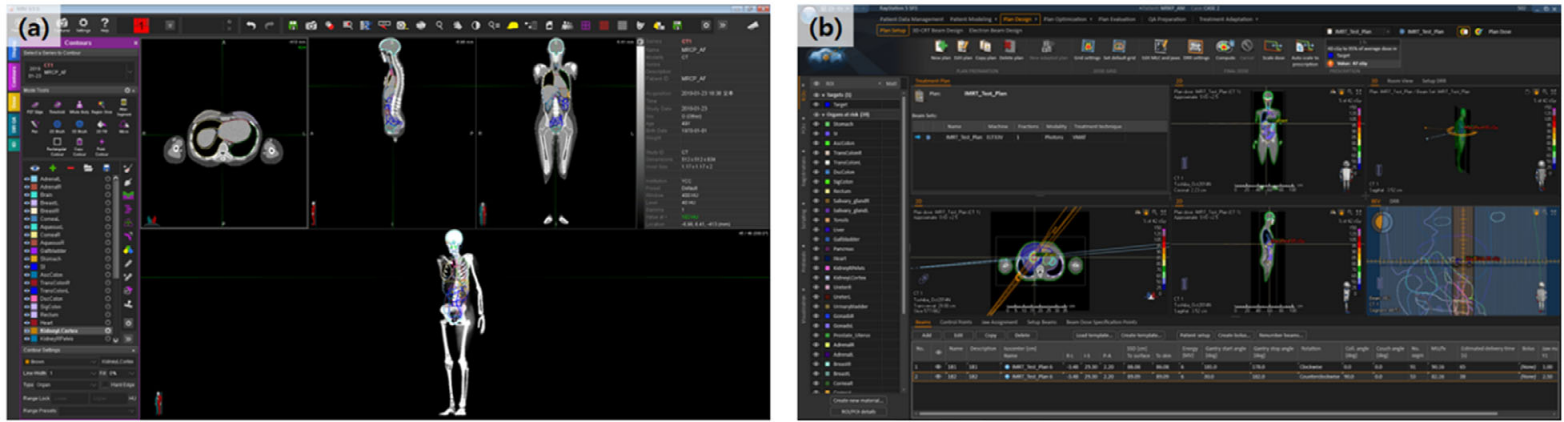
(a) MIM software with mesh‐type reference computational female phantom Digital Imaging and Communications in Medicine (DICOM) dataset and (b) RayStation5 with mesh‐type reference Korean male DICOM dataset

The produced DICOM datasets were imported to the commercial RT plan check system (MIM, MIM Software Inc., Cleveland, USA, version 6.5.6) and TPS (RayStation 5, RaySearch Laboratories, Stockholm, Sweden, version 5.0). Figure 5 shows that the DICOM dataset with the TM phantoms was successfully imported to the commercial systems. Notably, the TPS successfully established not only the MRCP DICOMs but also the MRKP DICOMs, as shown in Figure 5b.

### 3.3 Comparison of organ volumes between the TM phantom and the converted DICOM dataset

To evaluate the geometrical distortion of converted DICOM data quantitatively, volume comparison between a TM phantom and contour data converted by TET2DICOM was performed. Note that the major differences in geometrical distortion by TET2DICOM occurred from (1) resolution of points organizing contouring data and (2) slice thickness of the CT DICOM image, and the volume comparison could take into account both of the factors. In this study, the various organs of TM phantoms (brain, heart, lungs, colon, thyroid, adrenals, and corneas) were converted as a DICOM RT structure considering geometrical characteristics (e.g., size and complexity), and subsequently, each contouring volume was compared with that of a pertinent organ included in TM phantoms (Table 2). Note that the volumes of contouring data were calculated by using MIM Software.

**TABLE 2:** Comparisons of organ volume between tetrahedral‐mesh (TM) phantom and Digital Imaging and Communications in Medicine (DICOM) dataset

Organ	Phantom name	Organ volume		% diff. ([|A‐B|]/A) (%)
		TM phantom (A) (cm^3^)	DICOM dataset (B) (cm^3^)	
Brain	MRCP‐AM^1^	1 457.62	1 458.38	0.05
	MRCP‐AF^2^	1 296.41	1 297.06	0.05
	MRKP‐AM^3^	1 477.66	1 477.44	0.01
	MRKP‐AF^4^	1 389.10	1 389.03	0.01
Heart	MRCP‐AM^1^	848.24	848.59	0.04
	MRCP‐AF^2^	625.83	626.40	0.09
	MRKP‐AM^3^	799.46	799.84	0.05
	MRKP‐AF^4^	630.98	631.24	0.04
Lungs	MRCP‐AM^1^	2 888.52	2 888.94	0.01
	MRCP‐AF^2^	2 299.68	2 300.14	0.02
	MRKP‐AM^3^	4 633.18	4 633.90	0.02
	MRKP‐AF^4^	3 472.99	3 473.55	0.02
Colon	MRCP‐AM^1^	763.88	764.54	0.09
	MRCP‐AF^2^	742.27	742.63	0.05
	MRKP‐AM^3^	666.80	666.69	0.02
	MRKP‐AF^4^	526.71	526.77	0.01
Thyroid	MRCP‐AM^1^	22.22	22.30	0.36
	MRCP‐AF^2^	18.51	18.53	0.11
	MRKP‐AM^3^	14.28	14.27	0.07
	MRKP‐AF^4^	11.65	11.69	0.34
Adrenals	MRCP‐AM^1^	16.76	16.57	1.13
	MRCP‐AF^2^	14.94	15.12	1.20
	MRKP‐AM^3^	13.73	13.75	0.15
	MRKP‐AF^4^	13.00	12.99	0.08
Corneas	MRCP‐AM^1^	2.02	2.07	2.48
	MRCP‐AF^2^	2.02	1.98	1.98
	MRKP‐AM^3^	2.26	2.31	2.21
	MRKP‐AF^4^	2.26	2.28	0.88

^1^ Mesh‐type reference computational male phantom

^2^ Mesh‐type reference computational female phantom

^3^ Mesh‐type reference Korean male phantom

^4^ Mesh‐type reference Korean female phantom

The volume difference was less than 0.1% for most of the organs, while the corneas showed a difference of up to about 2.5%. The volume difference occurred due to interpolated volume between slices. The volume calculation increased when the size of the organs was small, because in those cases, curved surfaces or other complex geometry could not be represented with the CT voxels of the produced DICOM dataset (slice thickness); nevertheless, these tiny volume differences could be negligible in clinical practice because the maximum length of organ margin was <0.01 mm.

### 3.4 Conversion of deformed phantoms from TET2DICOM

**FIGURE 6:**
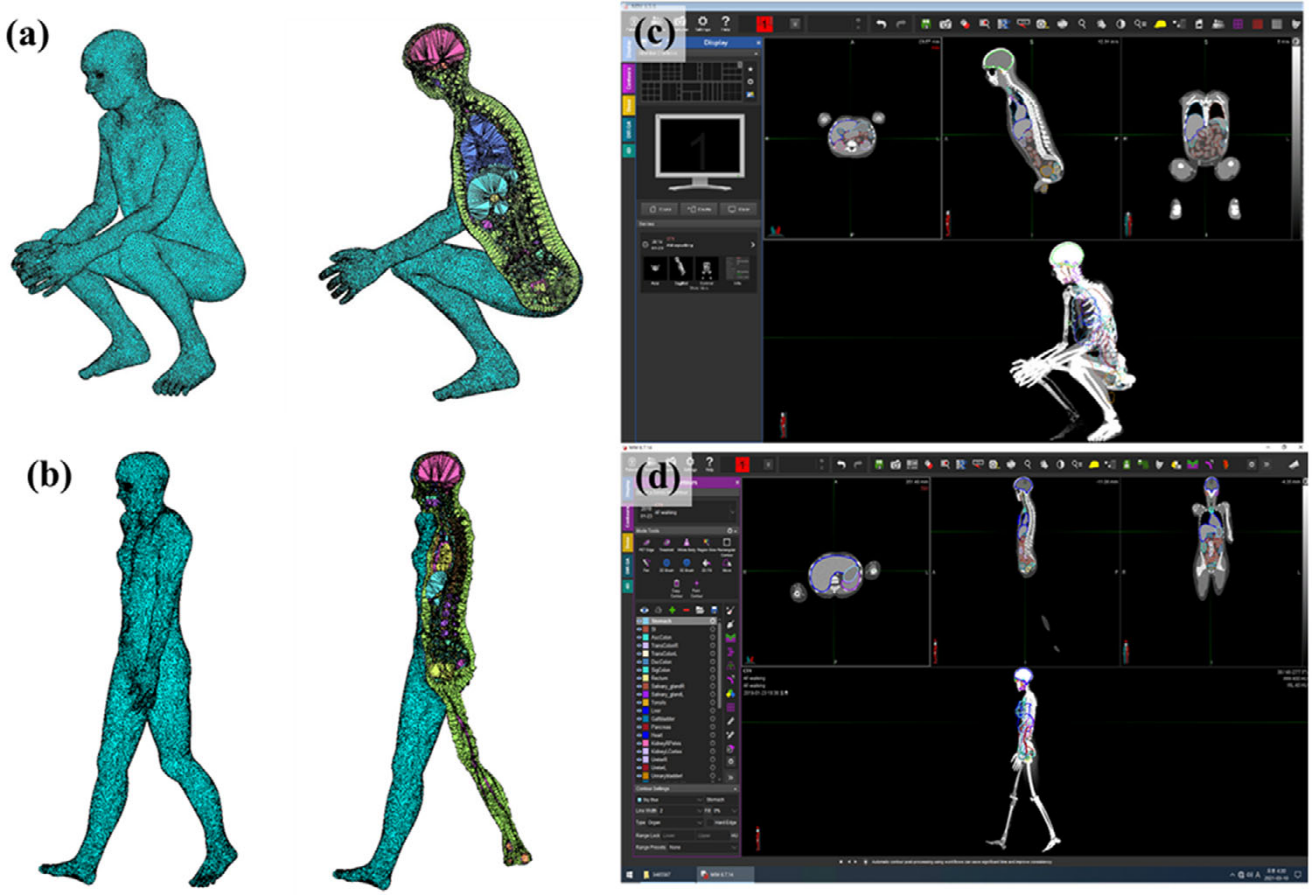
(a,b) Tetrahedral‐mesh phantoms in squatting and walking postures and (c,d) converted Digital Imaging and Communications in Medicine dataset implemented in MIM software

To evaluate the scalability of TET2DICOM that can be used in RT treatments that require special patient postures such as TBI or TSEBT,^32^, ^33^ the TM phantoms with different postures^22^ were converted to validate the converting process in various phantoms, such as patient‐specific phantom, and imported to clinical software. Figure 6 illustrates the TM phantoms in squatting and walking postures and (c,d) the converted DICOM dataset implemented in MIM software. As shown in Figure 6, the DICOM CT dataset was converted by TET2DICOM, and successfully implemented in the clinical software without any distortions. Note that all contoured volumes converted by TET2DICOM were calculated within a maximum difference of 0.5% comparing with those of their original organ volumes.

## 4 CONCLUSION

In this study, TET2DICOM was developed to produce a DICOM dataset for the TM phantom. TET2DICOM was evaluated by importing different types of reference TM phantoms to the clinical TPS. The TM phantoms were successfully converted into the DICOM‐RT dataset, which was also completely imported to clinical software. It can be used for MRKP/MRCP, which are used for performance verification, and for various types of recently developed TM phantoms.^34^, ^35^ Moreover, a TM phantom in various postures was successfully applied to the TPS. It is expected that TET2DICOM will prove effective in producing DICOM datasets for phantoms in various postures and that it will henceforward be well implemented to consider various patients’ somatotypes and postures in treatment planning. In addition, the TM phantom can directly calculate the dose in the MC simulation without any other process. We also believe that TET2DICOM could be used in various studies through comparison of dose results at TPS using DICOM and dose results using MC simulation. Unfortunately, TET2DICOM's application in clinical practice currently has limitations. The program currently operates with a character‐based user interface, and its conversion process takes a significant amount of time (approximately 120 min for MRCP‐AM). In the near future, we anticipate that this limitation will be addressed in program updates incorporating more powerful graphical user interface–based parallelization techniques.

## AUTHOR CONTRIBUTIONS

Bo‐Wi Cheon and Se Hyung Lee designed the program model and the computational framework and wrote the manuscript with input from all authors. Haegin Han, Chan Hyeong Kim, and Jin Sung Kim verified the conversion results for TPS implementation. Min Cheol Han and Chul Hee Min conceived the study and were in charge of the overall direction and planning. All authors discussed the results and contributed to the final manuscript.

## CONFLICT OF INTEREST

The authors have no conflict of interest to declare.
