# Erratum to Development of a novel program for conversion from tetrahedral‐mesh‐based phantoms to DICOM dataset for radiation treatment planning: TET2DICOM

**DOI:** 10.1002/acm2.14382

**Published:** 2024-06-10

Journal of Applied Clinical Medical Physics 23, 1 (January 2022) e13448

Bo‐Wi Cheon^1^, Se Hyung Lee^2,3^, Min Cheol Han^4^, Chul Hee Min^1^, Haegin Han^2^, Chan Hyeong Kim^2^, Jin Sung Kim^4^

^1^Department of Radiation Convergence Engineering, Yonsei University, Wonju, Korea

^2^Department of Nuclear Engineering, Hanyang University, Seoul, Korea

^3^Department of Radiation Oncology, Bundang Jesaeng General Hospital, Seongnam, Korea

^4^Department of Radiation Oncology, Yonsei University College of Medicine, Seoul, Kore

In the original publication of the article, on page 8, paragraph “Acknowledgements”, ‘Korea Institute of Energy Technology Evaluation and Planning (KETEP) grant funded by the Korea government (MOTIE) (G032579811).’ should change as ‘Korea Institute of Energy Technology Evaluation and Planning (KETEP) grant funded by the Korea government (MOTIE) (20214000000070).’

